# Intraspecific leaf trait variability along a boreal-to-tropical community diversity gradient

**DOI:** 10.1371/journal.pone.0172495

**Published:** 2017-02-27

**Authors:** Cristina C. Bastias, Claire Fortunel, Fernando Valladares, Christopher Baraloto, Raquel Benavides, William Cornwell, Lars Markesteijn, Alexandre A. de Oliveira, Jeronimo B. B. Sansevero, Marcel C. Vaz, Nathan J. B. Kraft

**Affiliations:** 1 Departamento de Biogeografía y Cambio Global. Museo Nacional de Ciencias Naturales- CSIC, Madrid, Spain; 2 Department of Biology. University of Maryland, College Park, MD, United States of America; 3 Department of Ecology and Evolutionary Biology, University of California, Los Angeles, CA, United States of America; 4 Área de Biodiversidad y Conservación, Universidad Rey Juan Carlos, Móstoles, Madrid, Spain; 5 INRA, UMR “Ecologie des Forets de Guyane”, Kourou Cedex 97387, French Guiana; 6 Institut für Biologie, Geobotanik, Albert-Ludwigs-Universität Freiburg, Freiburg, Germany; 7 Evolution & Ecology Research Centre, School of Biological, Earth and Environmental Sciences, University of New South Wales, Sydney, New South Wales 2052, Australia; 8 Smithsonian Tropical Research Institute, Balboa, Ancón, Panamá; 9 Department of Zoology, University of Oxford, South Parks Road, Oxford, United Kingdom; 10 School of Environment, Natural Resources and Geography, Bangor University, Bangor LL57 2DG, United Kingdom; 11 Departamento de Ecologia, Universidade de São Paulo, São Paulo, SP, Brasil; 12 Instituto de Pesquisas Jardim Botânico do Rio de Janeiro, Jardim Botânico, Rio de Janeiro, Brazil; 13 Universidade Federal Rural do Rio de Janeiro–UFRRJ. Departamento de Ciencias Ambientais–DCA. Instituto de Florestas–, Seropédica, Rio de Janeiro, Brazil; INRA—University of Bordeaux, FRANCE

## Abstract

Disentangling the mechanisms that shape community assembly across diversity gradients is a central matter in ecology. While many studies have explored community assembly through species average trait values, there is a growing understanding that intraspecific trait variation (ITV) can also play a critical role in species coexistence. Classic biodiversity theory hypothesizes that higher diversity at species-rich sites can arise from narrower niches relative to species-poor sites, which would be reflected in reduced ITV as species richness increases. To explore how ITV in woody plant communities changes with species richness, we compiled leaf trait data (leaf size and specific leaf area) in a total of 521 woody plant species from 21 forest communities that differed dramatically in species richness, ranging from boreal to tropical rainforests. At each forest, we assessed ITV as an estimate of species niche breadth and we quantified the degree of trait overlap among co-occurring species as a measure of species functional similarity. We found ITV was relatively invariant across the species richness gradient. In addition, we found that species functional similarity increased with diversity. Contrary to the expectation from classic biodiversity theory, our results rather suggest that neutral processes or equalizing mechanisms can be acting as potential drivers shaping community assembly in hyperdiverse forests.

## Introduction

The relative importance of ecological factors in shaping plant communities across species diversity gradients is the subject of longstanding debate in ecology [1–4] that has been recently invigorated by the lens of functional trait diversity [5,6]. The use of traits in a community ecology context hinges on the hypothesis that there is a link between traits and the breadth and position of species’ realized niches [7,8]. Trait-based studies have often used a trait mean approach (i.e. assigning all conspecific individuals a species average trait value) to examine community assembly mechanisms [9,10]. The implicit assumption in many of these studies is that interspecific trait differences are much larger than intraspecific trait differences [11,12]. However, there is increasing evidence that community assembly at local scales depends critically on the extent of intraspecific trait variation (ITV) [13–16]. Recently, the scientific community has reconsidered the importance of ITV [17–19] and its non-negligible contribution to the total trait variability, being sometimes as important as interspecific trait variation [20,21]. Even when interspecific trait differences are larger, incorporating ITV can improve the answer to key questions about the assembly and functioning of plant communities [15,22].

**Fig 1:**
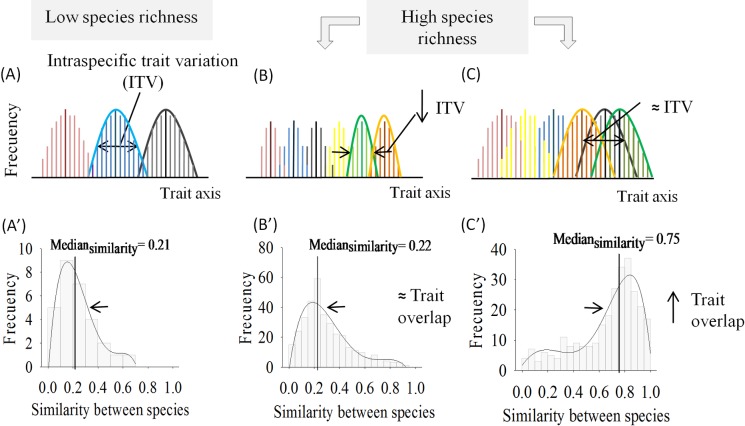
A schematic of possible changes in the extent of ITV and the degree of trait overlap (measured as the similarity between species; [32]) when species richness increases assuming a flat fitness landscape. (A) A substantial extent of ITV is expected in species-poor communities without increasing interspecific interactions since the biotic pressure is low. Therefore, low values of trait overlap are expected in species-poor communities leading to a right-skewed distribution (A’). (B) The extent of ITV is reduced, remaining trait overlap fairly constant to accommodate more species. It translates into a right-skewed distribution to low trait overlap values in species-rich forests (B’). (C) Trait overlap is increased without a change in the extent of ITV, resulting in greater degree of functional similarity among individuals. A left-skewed distribution of trait overlap values would be expected in species-rich forests (C’).

The extent of ITV among species in a community is expected to vary depending on community attributes such as the number of co-occurring species or the community trait diversity [13,19]. Previous studies have suggested that ITV should be greater in species-poor than species-rich communities [23,24]. If the biotic pressure via competitive interactions is lower in species-poor than in species-rich communities, conspecific individuals in species-poor communities could occupy a greater extension of available trait space (i.e. substantial extent of ITV) without increasing interspecific interactions (Fig 1A and 1A’). As the number of co-occurring species increases under the assumption of all co-occurring species with equal fitness (i.e. flat fitness landscape), species’ trait breadths are expected to be reduced (i.e. decline of ITV) to accommodate more species without increasing the potential for interspecific competition by resource use, consistent with classical niche theory [19,25] (Fig 1B). However, if the extent of ITV does not change from species-poor to species-rich communities or even increases (for example, see ‘individual variation’ theory by Clark [26]) and the trait range within a community in turn does not increase when species richness increases (Fig 1C), species’ trait overlaps would be expected to increase in more diverse communities (Fig 1C’). If traits map to resource use (and stabilizing niche differences, sensu Chesson [27]), species can coexist more readily by being functionally distinct, thereby promoting trait dissimilarity among species for coexistence [25]. However, some have argued that the lack of interspecific dissimilarity could lead to neutral dynamics (i.e. all individuals are considered functionally equivalent), reducing or removing the role of niche differences in shaping community assembly outcomes [28,29], or alternatively, communities structured primarily by the acting of equalizing mechanisms in a non-neutral model [30]. On the other hand, under an alternative niche differentiation model, specifically if species are differentiating along a landscape represented by combinations of peaks (high-fitness) and deep valleys (low-fitness) (i.e. multi-peak fitness landscape), a decrease of ITV is not predicted with species richness [31] despite a limiting similarity principle playing a role to select species on each peak.

While the relationships between species richness, ITV and trait overlap have important implications for community assembly and the maintenance of species richness [33], they have been poorly studied due to the substantial effort required to measure ITV, especially in species-rich systems [34]. Moreover, the few studies testing these relationships so far have shown contrasting results. For instance, some studies have found a decrease in the extent of ITV in specific leaf area (SLA) accompanied by declining SLA overlap with increased species richness [35,36]; whereas others have found no change in ITV in SLA or an increase of ITV and overlap using multiple approach (included SLA) with species richness [37]. These contradictory results show evidence that there is a need to improve our understanding of these relationships between species richness, ITV and trait overlap, exploring all possible approaches for a given trait in combination with the attributes of the study system in order to infer community assembly mechanisms across diversity gradients.

Here, we explore how the extent of ITV and trait overlap changes across a broad species richness gradient. We compile leaf trait data for 521 woody plant species found in 21 forest communities that varied in species richness from 1 to 284 species per hectare (from boreal and temperate to tropical forests). In accordance with the classic niche theory under a flat fitness landscape scenario, we hypothesize that the extent of ITV will decrease when more species are added to the community (Fig 1B) [25,27,38]. By extension, this hypothesis would suggest a reduction of the contribution of local intraspecific variation to the total trait diversity of the community with increasing species richness. Alternatively, if ITV does not decline and the overall length of the trait gradient does not increase in diverse communities, we expect a higher overlap in trait distributions among species with increasing species richness (Fig 1C). This would translate into a left-skewed distribution of trait overlap values, i.e. higher median values of trait overlap as well as a higher proportion of pairs of species with high trait overlap in species-rich than species-poor forests (Fig 1C’). By extension, ITV could be expected to contribute to the total trait diversity similarly or even more than interspecific trait differences in rich communities.

## Materials and methods

### Data collection

We used two leaf traits to quantify ITV: leaf size refers to individual leaf area (cm^2^) and specific leaf area (SLA), defined as the ratio of leaf area to dry mass (cm^2^ · g^-1^) [39]. We focused on these traits, firstly, because different empirical studies of woody plants from a wide range of environments have shown that SLA and leaf size are weakly or not correlated across species or at species level [40,41]. Secondly, these key leaf traits have been widely used in species distribution studies across gradients to predict future shifts in individual species distributions or even in species-realized niches due to their strong response to abiotic and biotic changes [41,42]. Moreover, SLA and leaf size are traits with an important ecological significance in relation to plant economics and plant resource acquisition: plants investing in greater SLA values increase light-capture efficiency, but are more vulnerable to high temperatures, drought, nutrient-limitation and herbivory [43]. On the other hand, leaf size is more related to the thermal conductance of the leaf boundary layer. Smaller leaf size helps to keep optimal leaf temperature and a higher water balance efficiency, especially under high solar radiation and low water availability conditions [44]. Finally, these traits can easily be measured on a large number of individuals.

We measured leaf traits in 3712 individuals of 521 woody plant species in 21 forest communities with contrasting species richness, ranging from the species-poor boreal and temperate European forests (1–4 species/ha) to hyperdiverse rainforests in Ecuador and Brazil (>200 species/ha) (Table 1). Species richness was calculated for each forest as the number of species with a diameter at breast height (DBH) ≥ 10 cm as it is customary [45–48]. We also obtained climatic variables (the mean annual temperature and annual precipitation) per each forest community from the Worldclim global climate models [49]. We included leaf trait data for species from a given community using the following criteria: (i) at least five individuals per species were measured in each forest in order to estimate ITV (see [50]), but it was higher whenever was possible (Table 1). (ii) To minimize the influence of ontogenetic variation on ITV, we restricted our sampling to understory individuals (saplings and small-stature trees), whose DBH is smaller than 20 cm and height less than 10 m. To restrict the sampling to understory individuals may imply either an underestimation or overestimation in the number of sampled species compared to the species richness of the community (calculated taking into account individuals with a DBH >10cm). An underestimation may occur since we are sub-sampling woody species from a forest layer, but also an overestimation since the arrival of propagules from neighbor canopies can lead to new established individuals in the understory of the canopy of the focal community. (iii) Due to the considerable effort required in sampling at ITV level in hyperdiverse forests, we only conducted trait measurements on a subset of the common species in tropical forests (those species that we found at least 5 individuals) (see Table 1 for the number of sampled species in each forest). This restriction in tropical forests may result in an underestimation of the extent of ITV and trait overlap among species in these forests since we do not have trait data available for the whole range of naturally occurring trait variation (i.e. we do not consider those rare species that occupy unique trait space compared to common species [51,52]). Besides, common species could show less trait variation [44], which further contributes to underestimating actual ITV. (iv) Also, it is important to mention that trait data used here were previously collected for other specific goals (see Table 1 for original references), but in general, individuals and leaves were chosen under standardized abiotic conditions (i.e. recently matured and fully expanded leaves) [39]. As a consequence, we may underestimate the actual ITV since ITV measured here is controlled for two main sources of variation: phenotypic plasticity in response to local abiotic conditions (i.e. we biased ITV towards natural standardized conditions) and ontogenetic variations (i.e. we biased ITV at a single ontogenetic stage: understory individuals). Overall, all criteria were consistently in direction of the underestimation of actual ITV.

**Table 1:** Description of each forest community included in the study.

Type of forest	Location	Latitude	Longitude	Mean annual T^ra^ (°C)	Pp (mm)	SR	Representative families of sampled species	No. species measured with ≥ 5 individuals	Individuals measured per species (Min, Max)	Original references
Boreal forest	Joensuu, North Karelia (Finland)	62.616	29.89	2.1	628	1	Pinaceae, Betulaceae	3	[19, 55]	(Bastias *et al*. unpublished data)
Boreal forest	Joensuu, North Karelia (Finland)	62.504	29.76	2.1	628	2	Pinaceae, Betulaceae	2	[5, 64]	(Bastias *et al*. unpublished data)
Boreal forest	Joensuu, North Karelia (Finland)	62.558	30.16	2.1	628	3	Pinaceae, Betulaceae	2	[7, 8]	(Bastias *et al*. unpublished data)
Mountainous beech forest	Carpathian mountains (Romania)	47.295	26.05	5.6	689	1	Pinaceae, Fagaceae, Sapindaceae	4	[11, 54]	(Bastias *et al*. unpublished data)
Mountainous beech forest	Carpathian mountains (Romania)	47.294	26.05	5.6	689	2	Pinaceae, Fagaceae, Sapindaceae	4	[8, 60]	(Bastias *et al*. unpublished data)
Mountainous beech forest	Carpathian mountains (Romania)	47.292	26.05	5.6	689	3	Pinaceae, Fagaceae, Sapindaceae	3	[10, 37]	(Bastias *et al*. unpublished data)
Mountainous beech forest	Carpathian mountains (Romania)	47.291	26.05	5.6	689	4	Pinaceae, Fagaceae, Sapindaceae	2	[5, 20]	(Bastias *et al*. unpublished data)
Mediterranean mixed forest	Alto Tajo Natural Park (Spain)	40.731	-2.25	9.9	533	1	Pinaceae, Fagaceae	4	[6, 57]	(Bastias *et al*. unpublished data)
Mediterranean mixed forest	Alto Tajo Natural Park (Spain)	40.713	-2.19	9.9	533	2	Pinaceae, Fagaceae	4	[21, 70]	(Bastias *et al*. unpublished data)
Mediterranean mixed forest	Alto Tajo Natural Park (Spain)	40.698	-2.13	9.9	533	3	Pinaceae, Fagaceae	2	[5, 10]	(Bastias *et al*. unpublished data)
Tropical lowland dry deciduous forest	Inpa, Concepcion, Santa Cruz (Bolivia)	-16.117	-61.72	23.5	1124	34	Fabaceae, Flacourtiaceae, Euphorbiaceae	52	[5, 10]	[48]
Riparian, chaparral, broadleaf evergreen forest	Jasper Ridge Biological Preserve (California, USA)	37.4	-122.25	13.8	598	54	Fagaceae, Rosaceae, Rhamnaceae	43	[5, 42]	[42]
Tropical lowland semi-deciduous seasonal moist forest	Soberania National Park (Panama)	9.162	-79.75	26	2553	131	Fabaceae, Piperaceae, Rubiaceae	16	[5, 6]	(Markesteijn, unpublished data)
Lowland tropical rainforest	Acarouany (French Guiana)	5.544	-53.81	26.5	2237	148	Annonaceae, Burseraceae, Lecythidaceae	11	[3, 22]	[10,53]
Lowland tropical rainforest	Paracou (French Guiana)	5.272	-52.93	25.8	2821	150	Euphorbiaceae, Fabaceae, Lecythidaceae	35	[3, 39]	[10,53]
Lowland tropical rainforest	BAFOG (French Guiana)	5.494	-53.99	26.4	2460	156	Annonaceae, Burseraceae, Lecythidaceae	11	[3, 25]	[10,53]
Lowland tropical rainforest	Nouragues (French Guiana)	4.087	-52.67	24.8	3337	197	Lecythidaceae, Malvaceae, Sapotaceae	24	[3, 25]	[10,53]
Lowland tropical rainforest	Montagne Tortue (French Guiana)	4.219	-52.41	24.6	3591	213	Sapotaceae	14	[3, 11]	[10,53]
Lowland tropical rainforest	Saut Lavilette (French Guiana)	4.151	-52.2	25.7	3590	224	Annonaceae, Sapotaceae	10	[3, 28]	[10,53]
Evergreen lowland tropical rainforest	Yasuní National Park (Ecuador)	0.683	-76.4	25	3129	251	Euphorbiaceae, Annonaceae, Fabaceae, Myrtaceae	59	[3, 21]	[9]
Lowland tropical rainforest	Biological Dynamics of Forest Fragments Project Reserve (BDFFP). Florestal and Cabo Frio (Central Amazon, Brazil)	-2.433	-59.83	27	2410	284	Fabaceae, Lecythidaceae, Sapotaceae	16	[3, 7]	[54] Oliveira, unpublished data

Pp: annual precipitation. SR: number of species with a DBH ≥10cm /ha.

Sampling permissions were granted by Delegación Provincial de la Conserjería de Agricultura y Medio Ambiente (Guadalajara-Castilla La Mancha) for the Mediterranean mixed forest in Alto Tajo Natural Park (Spain), by Instituto Boliviano de Investigación Forestal (IBIF) and the logging companies INPA Parket Ltd. and Planet La Chonta Investment Ltda for the tropical lowland dry deciduous forest in Bolivia, by Ministerio de Ambiente de Panama (MiAmbiente) for the tropical lowland semi-deciduous seasonal moist forest in Panama, by ICMBio/SISBIO- license number 23191–1 for the lowland tropical Atlantic Forest (Poço das Antas Biological Reserve–Southeastern Brazil), by Ministerio del Ambiente of Ecuador for the evergreen lowland tropical rainforest in Yasuní National Park (Ecuador) and by ICMBio/SISBIO- license number 18757–1 for the lowland tropical rainforest in Central Amazon (Brasil). For the rest of forests were not required specific permissions. The authorities responsible of these areas were informed and they expressed their consent to this sampling. Moreover, sampling did not involve endangered or protected species.

### Statistical analyses

We used the coefficient of variation (CV; 100 * standard deviation / mean) as an estimate of ITV. Because ITV may be influenced by the number of individuals sampled, we performed a rarefaction analysis in order to account for differences in sample size among species within and among forest communities [55]. This rarefaction analysis generated an expected trait value for each species in each forest by randomly drawing five individuals from the total pool of individuals of each species. We repeated this re-sampling process 1000 times for each species in each forest community. We then calculated the CV for each species from the average of the expected trait values generated by 1000 randomizations. To be sure of unbiased statistics estimated from rarefaction analysis, we checked both the community’ rarefaction curves did not cross (S1 Fig) and also, species ranks in ITV were the same across sample sizes (S2 and S3 Figs)[55]. Moreover, we confirm that species with smaller sample sizes did not have systematically lower ITV values (S4 Fig for leaf size and S5 Fig for SLA). We performed a generalized linear mixed model (GLMM, [56]) using ITV as response variable, species richness as an explanatory variable together with the mean annual temperature and annual precipitation as explanatory covariates in order to account for climate differences among forests. Type of forest was included as a random factor to control for other intrinsic characteristics of each community.

We also assessed the degree of trait overlap among co-occurring species and its relationship with species richness. Trait overlap is defined as the overlapping area between two trait distribution curves [32] and calculated by (1) assuming that trait values of a species are normally distributed around the mean [25,57] or (2) using kernel density estimators, which do not assume any particular shape of the trait distribution [58]. Using a normal distribution rather than kernel density tends to overestimate trait overlap, but at the same time it is considered more robust to small sample size (i.e. in our case, species with 5 individuals) than kernel distribution [58]. Because of these concerns, we estimated trait overlap with both normal and kernel density approaches using the R function Trova [32]. For analyses with normal distributions, a mean and standard deviation of the traits is required for each species in each forest community. Given the differences among species in sampling intensity, we first ran a rarefaction analysis for each species by randomly re-sampling 5 individuals per species per site, repeated 1000 times. We then calculated the mean and standard deviation from the average of expected trait values from 1000 randomizations for each species and forest community. Trait overlap figures for both methods range from 0 to 1, where values close to 1 indicate a high overlap between species or a high trait similarity. We calculated the median and the proportion of values obtained with low (less than 0.25 out of 1) and high trait overlap (higher than 0.75 out of 1) as a categorical description of the distribution of trait overlap values for each community from both methods. Finally, we applied linear regression models using categorical parameter description of the distribution of trait overlap values as response variables and species richness as explanatory variable. Categorical parameters were square root transformed to improve normality.

All statistical analyses were carried out in R v. 3.2.1 [59] using the packages lme4 [60] and MuMIn [61].

## Results

### Intraspecific trait variability and species richness

**Fig 2:**
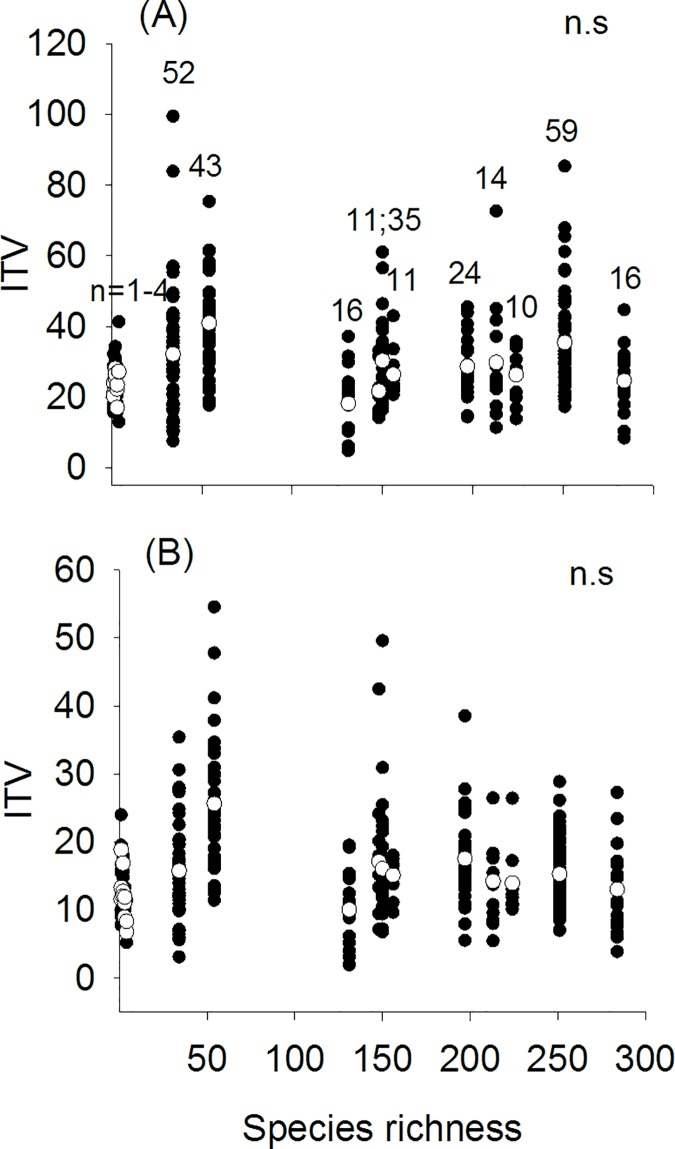
**Effect of species richness on the extent of ITV (estimated as coefficient of variation, CV) for (A) leaf size and (B) SLA.** n = number of species measured for each forest community with ≥ 5 individuals measured. Empty circles indicate the average of ITV values for each forest community and richness level. The number of sampled species (n) may be both lower than the species richness of the community since we sub-sampled a forest layer, but also, potentially larger than the species richness of that community since the arrival of seeds from neighbor canopies may result in the establishment of new individuals that previously were not part of the main canopy of the focal community.

We found considerable ITV for both leaf traits among species co-occurring in all forest communities (Fig 2A and 2B). Accordingly, ITV for leaf size and SLA did not vary consistently with species richness (Fig 2A and 2B; Table 2). None significant effects were also observed for climatic covariates (mean annual temperature and annual precipitation) on the extent of ITV for both studied traits (Table 2).

**Table 2:** Summary table of the GLMM testing the change in the extent of ITV with species richness (SR), mean annual temperature (Mean. Ann. T^ra^) and annual precipitation (Pp) for both leaf size and SLA.

Response	Exp. variable	Estimate	SD. Error	t-value	p-value	R^2^_m_	R^2^_c_
Leaf size n = 321	Intercept	5.098	0.379	13.436	0.000***	0.010	0.213
	SR	0.027	0.004	0.706	0.480		
	Mean. Ann. T^ra^	0.003	0.031	0.119	0.905		
	Pp	-0.000	0.000	-0.577	0.564		
SLA n = 315	Intercept	3.726	0.302	12.338	0.000***	0.009	0.243
	SR	0.001	0.003	0.469	0.639		
	Mean. Ann. T^ra^	0.013	0.025	0.512	0.459		
	Pp	-0.000	0.000	-0.740	0.609		

n = number of species included in the analyses; R^2^_m_: marginal-R^2^; R^2^_c_: conditional-R^2^.

(***): p-value <0.001

(**): p-value <0.01

(*): p-value < 0.05

(.): p-value <0.1.

### Trait overlap and species richness

**Fig 3:**
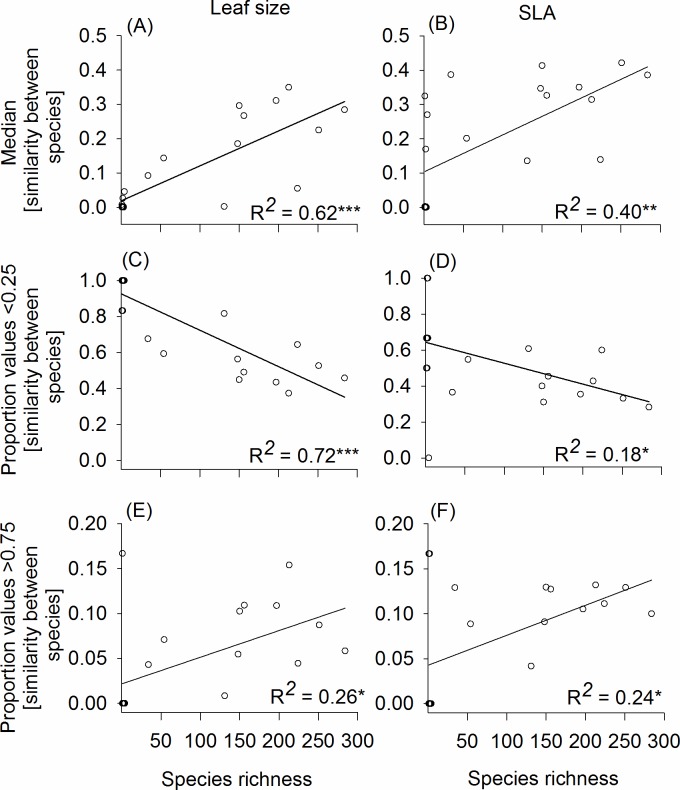
**Linear regression models of the median values of trait overlap (panel A, B) and the proportion of low (less than 0.25; panel C, D) and high (> 0.75; panel E, F) values of degree of trait overlap between species for each forest community against species richness for both leaf size (left) and SLA (right).** Trait similarity was calculated by assuming normal trait distribution of species with ≥ 5 individuals measured. (***): p-value <0.001; (**): p-value <0.01; (*): p-value < 0.05; (.): p-value <0.1.

For both leaf traits, median trait overlaps between species for each forest were significantly lower when assuming normal trait distribution than using kernel density estimators (Wilcoxon-Signed Rank test: n = 21; Z = 3.7; p-value < 0.001 for leaf size; n = 21, Z = 3.8, p-value < 0.001 for SLA). We found increasing trait overlap with species richness for both leaf traits using normal trait distribution (Fig 3A and 3B). We obtained similar results using kernel density estimators (r^2^ = 0.64, p-value < 0.001 for leaf size, S6A Fig; and r^2^ = 0.44, p-value < 0.001 for SLA, S6B Fig). We found that the proportion of species pairs with very low trait overlap (< 0.25) decreased significantly with species richness for both traits assuming normal trait distribution (Fig 3C and 3D) and kernel density approximation (r^2^ = 0.72, p-value < 0.001 for leaf size, S6C Fig and r^2^ = 0.27, p-value < 0.001 for SLA, S6D Fig). Moreover, we found that the proportion of species pairs with high trait overlap (> 0.75) increased significantly with species richness for both traits assuming normal distribution (Fig 3E and 3F). However, under kernel approach, the relationship between the proportion of species pairs with high trait overlap with species richness was only marginally significant for leaf size (r^2^ = 0.14, p-value = 0.07 for leaf size, S6E Fig) and none relationship was observed for SLA (S6F Fig).

## Discussion

A largely unanswered question in biodiversity theory is whether ITV actually varies with species richness as predicted by classical niche theory [19,25] assuming all co-occurring species with equal fitness, that postulates that species would show narrower trait breadths (i.e. decrease of ITV) with increased species richness in order to avoid competition. Contrary to niche theory, our results showed a lack of relationship between ITV and species richness for leaf size and SLA, suggesting that the species’ niches did not exhibit tight packing of the trait space in species-rich forests. In addition, we found greater trait overlap in species-rich communities for both traits, reflected here by an increase of the median values in trait overlap, with decreasing proportion of species pairs with low trait overlap and thus increasing proportion of species pairs with high trait overlap as species richness increased. Our results did not support the predictions from the principle of limiting similarity, which predicts a higher spread of trait values (i.e. trait dissimilarity among species) at the community level [42,62] since co-occurring individuals with high similarity in ecological requirements are more likely to face competitive exclusion [25].

The few studies testing the species richness-ITV-trait overlap relationships have so far focused on a single study system, plant growth form or functional trait, and found inconsistent results [35–37]. Some studies have shown declines in ITV and reduction of trait overlap with increasing species richness. For example, Hulshof *et al*. [35] found that the ratio between intraspecific and interspecific variability (a good proxy of trait overlap among species [19,32]) in SLA in woody plant communities decreased with increasing species richness. A similar pattern was also found by Kumordzi *et al*. [36] when studying variation in SLA of understory vegetation across different boreal forest communities differing in species diversity. Felten *et al*. [63] found a decrease of niche breadth and niche overlap in temperate grasslands with increasing species richness, indicating complementarity of soil N use from different soil depth. In contrast, others have shown increasing ITV and trait overlap with increasing species richness, in concordance with our findings. For example, using a multi-trait approach (including SLA) in limestone grasslands, Le Bagousse-Pinguet *et al*. [37] found increases in both ITV and the ratio between intraspecific and interspecific variability with increasing species richness, with no effect of environment on ITV in agreement with our results. They suggest asymmetric light competition among competing species as a potential explanation to this pattern. It may merely be due to the increased probability of having individual plants from a shade-intolerant species being slightly more tolerant than an individuals of a supposed shade-tolerant species; this situation would induce ITV and would minimize the differences in plant fitness and competitive ability among co-occurring species [36]. Finally, a recent global meta-analysis Siefert *et al*. [16] reports that the relative extent of ITV to the total community trait variance decreases with increasing species richness, but this pattern is mainly due to an increase in interspecific variance and consequently, in the total community variance, whereas the absolute extent of ITV remains fairly constant with species richness. While the analysis from Siefert *et al*. [16] supports partly our results, it includes different plant growth forms (i.e. both herbaceous and woody plants) from multiple community types (from grasslands to forests). Differences in ITV between plant forms can be expected since long-lived woody plants may present limited plasticity (i.e. less ITV) due to higher investment in longer lifespan tissues over their lifetimes compared to short-lived herbaceous species. Unlike, our study is based on a single growth form (freestanding woody plants in forest communities) on which debates about community assembly processes across the latitudinal gradient have been especially focused in recent years (e.g. [4,28,29,33,64,65]). It is important to note that a different way to calculate the ITV is used in our study in comparison with these aforementioned studies. Whereas our study measures ITV as the coefficient of variation at individual species level (i.e. absolute ITV at species level), others measure the mean intraspecific trait variance at community level (including ITV of all coexisting species in relation to total community trait variance). This difference in ITV measurement could explain in somehow differences in patterns found with respect to our results [16,35].

Our findings showing an increased functional similarity in hyperdiverse forests suggest, as Hubbell and Chave have argued (e.g. [28,29,66]), that it is more likely that individuals in high diversity forests are more functionally similar to each other than individuals in lower diversity forests. On the one hand, this can lead to a greater chance for neutral or nearly neutral dynamics in these more diverse communities [29]. On the other hand, if a higher degree of functional similarity between species pairs in diverse forests translates into smaller fitness differences between species (sensu Chesson [27]), only modest stabilizing niche differences between species (e.g. resource partitioning, density-dependent effects or population density fluctuations) would be required to drive community dynamics in a non-neutral fashion [38]. Unfortunately, these questions cannot be resolved without deeper understanding of how trait differences in woody plant communities relate to fitness and stabilizing niche differences. While recent experimental works have made these links for algal [67] and annual plant communities [68], considerable logistical barriers remain in long-lived plants as woody communities.

We suggest that our findings contradict niche theory and the principle of limiting similarity (promoting trait dissimilarity among species), but only based on previous assumptions of a flat fitness landscape (i.e. all species with equal fitness). However, an alternative conclusion could be reached assuming a multi-peak fitness landscapes [69]. Under multi-peak fitness landscapes, species on each peak may have been selected by the interplay of different processes among which competitive limit to similarity. In this scenario, species can reach equal fitness and maintain similar ITV [31] despite species richness increases (i.e. no decrease of ITV with species richness is expected).

In this study we explored shifts in the extent of ITV and trait overlap along a broad species richness gradient, but it is important to mention that our results may be in part limited since we did not carry out trait measurements on all individuals or species that were part of the whole community, particularly in species-rich communities. This likely may translates into an underestimation of the extent of ITV and trait overlap between species in diverse forests. Nevertheless, the high degree of species rarity in our tropical forests [70] makes it difficult to reach a complete range for ITV. Moreover, it is important to take into account that we are using two dimensions of the plant ecological strategy (SLA and leaf size) and they may not be good proxies of plant species’ realized niches in species-rich forests. In other words, they may not be capturing niche differences among species, being the competitive ability for limiting resource use determined by other key traits that we did not take into account. Further analyses incorporating other traits representative of different plant strategy axes, such as architecture traits and woody density, and even a multi-trait approach could improve our understanding about the traits that best relate to fitness and, thus, drive niche differentiation [70]. Furthermore, local biotic and abiotic factors also have effects on the extent of ITV by selecting a particular subset of trait values according to the local environment [71]. Future studies integrating other local environmental factors, such as crowding, light availability or water availability [72–74], as well as the environmental heterogeneity [75] would improve our understanding of the factors driving the relationship between ITV and species richness.

To conclude, our study highlights the key role that trait variability within species can play in understanding community assembly along biodiversity gradients, and emphasizes the value of estimating intraspecific variability in studies exploring trait diversity at the community level. We found an increase of functional similarity among co-occurring species in more diverse communities, to the widely recognized classical niche theory. Our study points to neutral processes or equalizing mechanisms to explain that species with similar ecological requirements can be present in the same community at the same time.

## Supporting information

S1 Dataset This file contains data belonging to the article "Intraspecific leaf trait variability along a boreal-to-tropical community diversity gradient" by Cristina C. Bastias, Claire Fortunel, Fernando Valladares, Christopher Baraloto, Raquel Benavides, William Cornwell, Lars Markesteijn, Alexandre A. de Oliveira, Jeronimo B.B Sansevero, Marcel C. Vaz, Nathan J. B. Kraft. The first sheet contains leaf trait data for each individual within each species and type of forest. The second sheet contains data from the rarefaction analyses: the rarefied mean, the rarefied coefficient of variation (CV; Fig 2) and the rarefied standard error (sd) for both leaf size (LS) and SLA at species level. Also, it contains the sample size (n) per each species and the species richness for each community (SR). The third sheet presents the overlap data assuming normal trait distribution for both leaf size (LS) and SLA: median, proportion of low (< 0.25) and high (> 0.75) values of degree of trait overlap between species per each type of forest (Fig 3). The last sheet presents the overlap data under a kernel approach for both leaf size (LS) and SLA: median, proportion of low (< 0.25) and high (> 0.75) values of degree of trait overlap between species per each type of forest (S6 Fig).
(XLSX)

S1 Fig Community accumulation curves at sample size of 5 individuals. Dashed lines are 95% confident.
(TIF)

S2 Fig Species ranks at sample size of 10 individuals per species (ITV) *vs* species ranks at sample size of 5 individuals (rarefied ITV) in order to detect bias in the ITV estimate by a small sample size. R^2^ close to 1 means no bias (i.e. similar ITV values obtained for a species using 10 individuals and using 5 individuals).
(TIF)

S3 Fig Species ranks at sample size of 20 individuals per species (ITV) *vs* species ranks at sample size of 5 individuals (rarefied ITV) in order to detect bias in the ITV estimate by a small sample size. R^2^ close to 1 means no bias (i.e. similar ITV values obtained for a species using 20 individuals and using 5 individuals).
(TIF)

S4 Fig Boxplot of the ITV values for leaf size grouped in 7 categories of sample size per species (No. of individuals). n: number of observations in each category.
(TIF)

S5 Fig Boxplot of the ITV values for SLA grouped in 7 categories of sample size per species (No. of individuals). n: number of observations in each category.
(TIF)

S6 Fig Linear regression models of the median values of trait overlap (panel A, B) and the proportion of low (less than 0.25; panel C, D) and high (> 0.75; panel E, F) values of degree of trait overlap between species for each forest community against species richness for both leaf size (left) and SLA (right). Trait similarity was calculated by kernel density approach using species with ≥ 5 individuals measured. (***): p-value <0.001; (**): p-value <0.01; (*): p-value < 0.05; (.): p-value <0.1.
(TIF)
