# Supplementary figures and images for: Intraspecific leaf trait variability along a boreal-to-tropical community diversity gradient
Source: PLoS One. 2017 Feb 27;12(2):e0172495. doi: 10.1371/journal.pone.0172495 (PMC5328268; doi:10.1371/journal.pone.0172495)

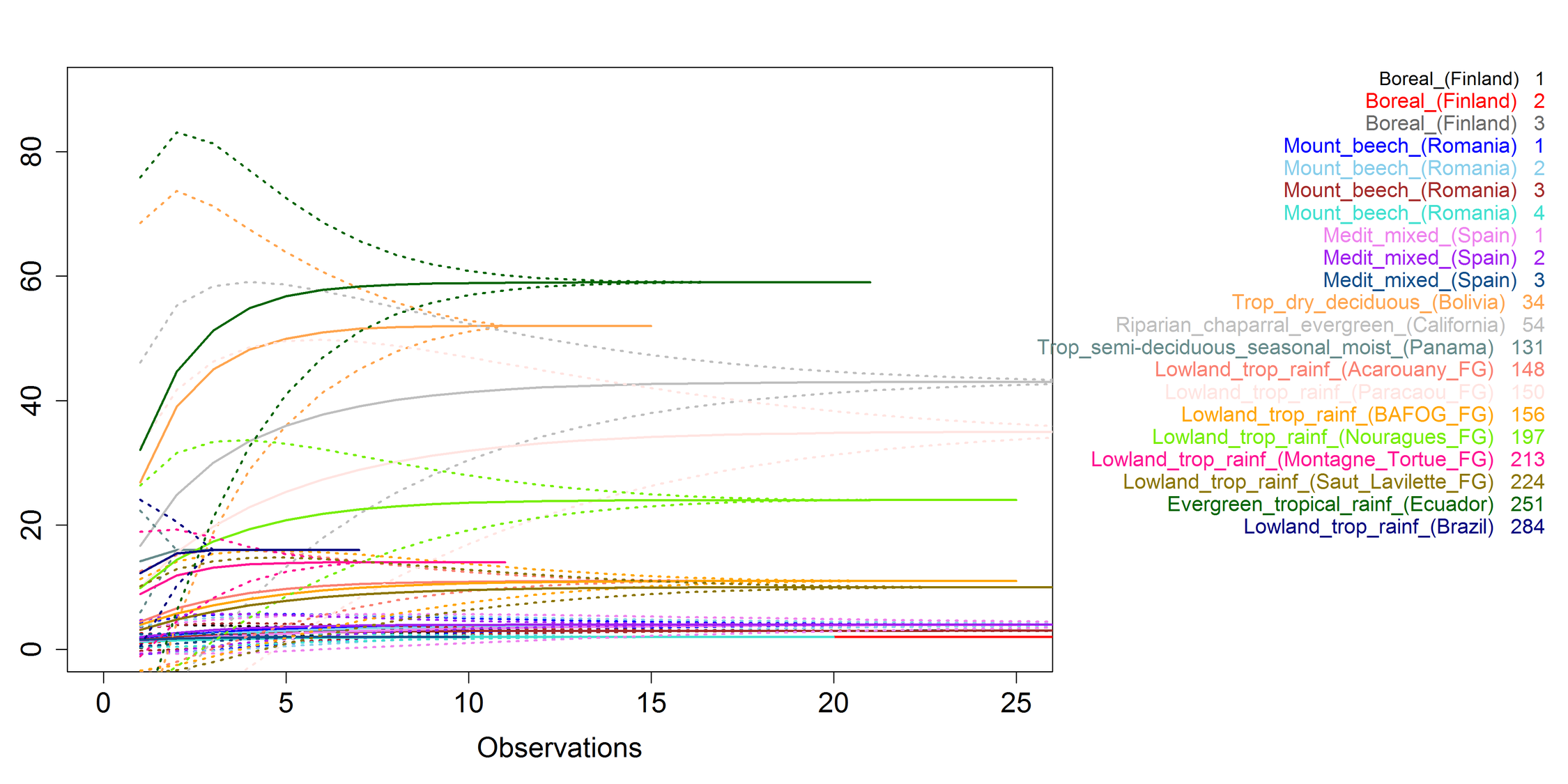

Supplement: S1 Fig — Dashed lines are 95% confident. (TIF) [file pone.0172495.s002.tif]

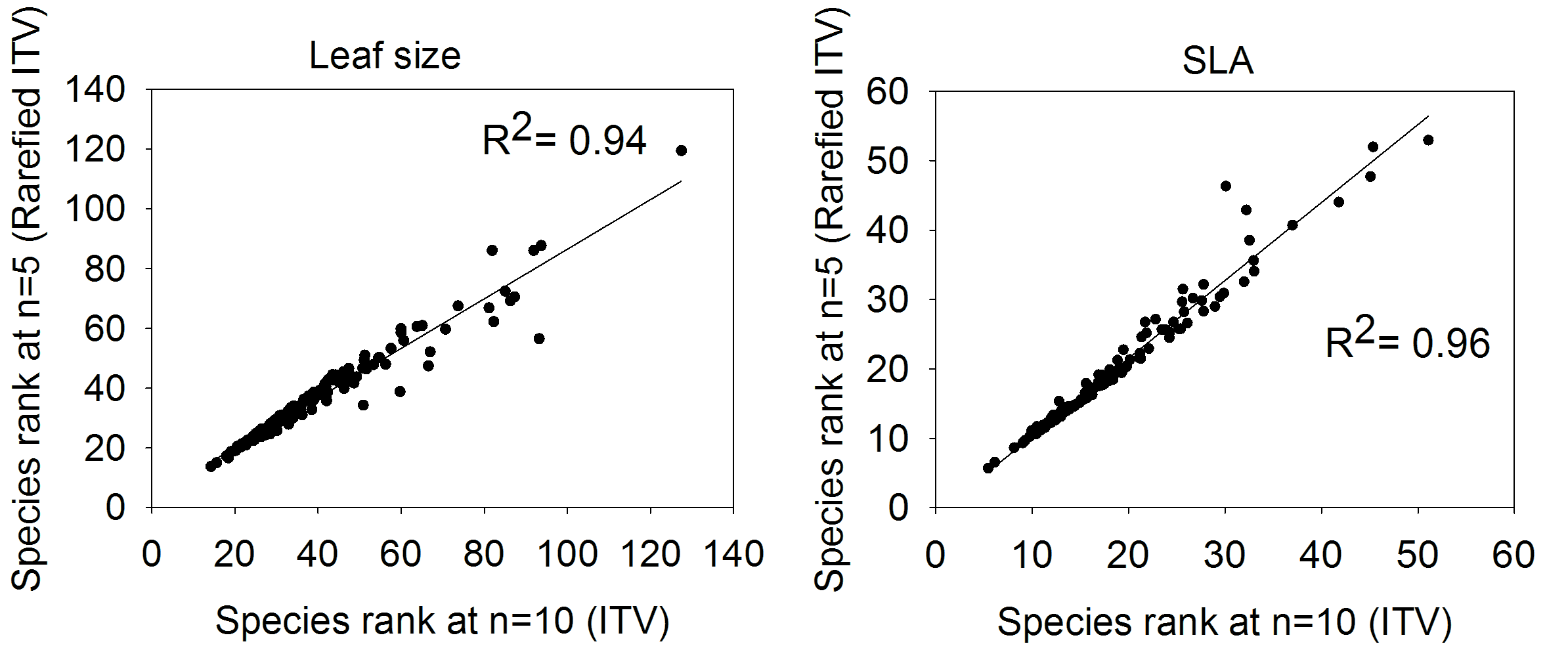

Supplement: S2 Fig — R2 close to 1 means no bias (i.e. similar ITV values obtained for a species using 10 individuals and using 5 individuals). (TIF) [file pone.0172495.s003.TIF]

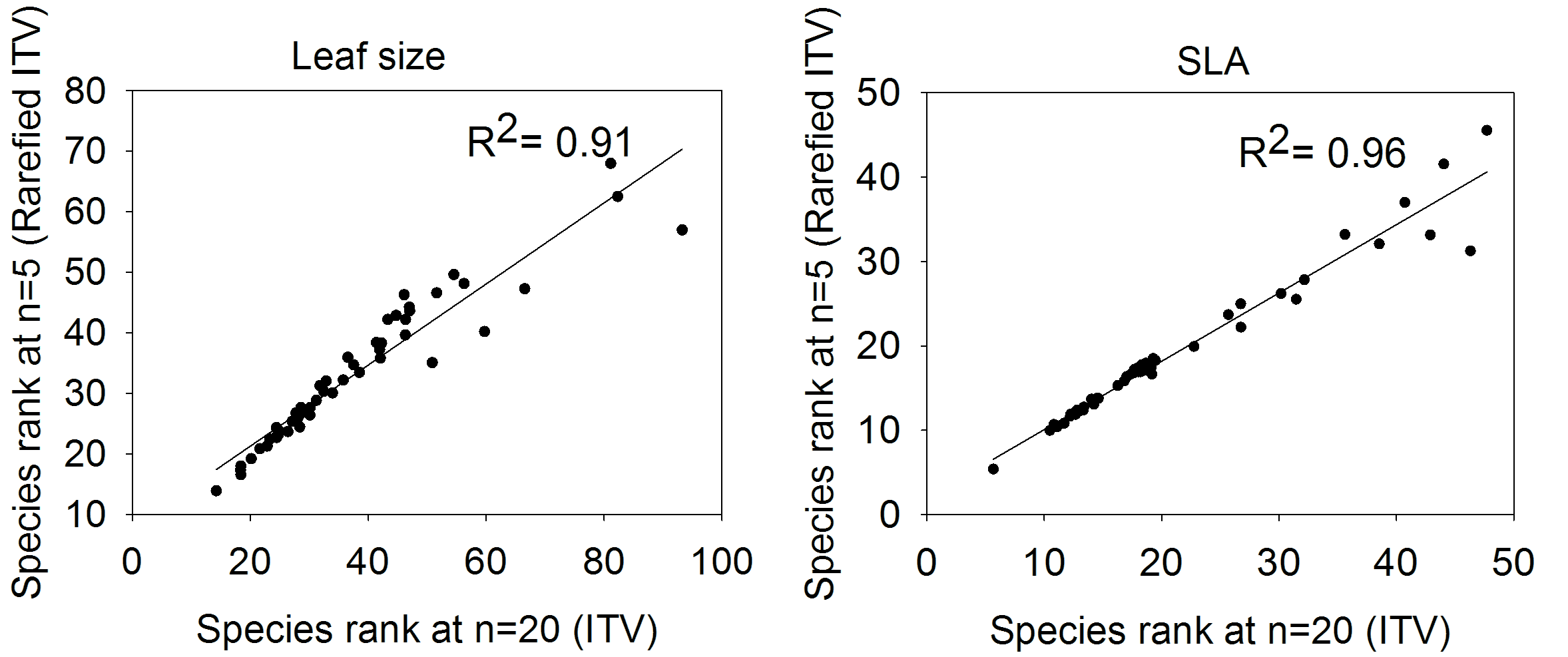

Supplement: S3 Fig — R2 close to 1 means no bias (i.e. similar ITV values obtained for a species using 20 individuals and using 5 individuals). (TIF) [file pone.0172495.s004.TIF]

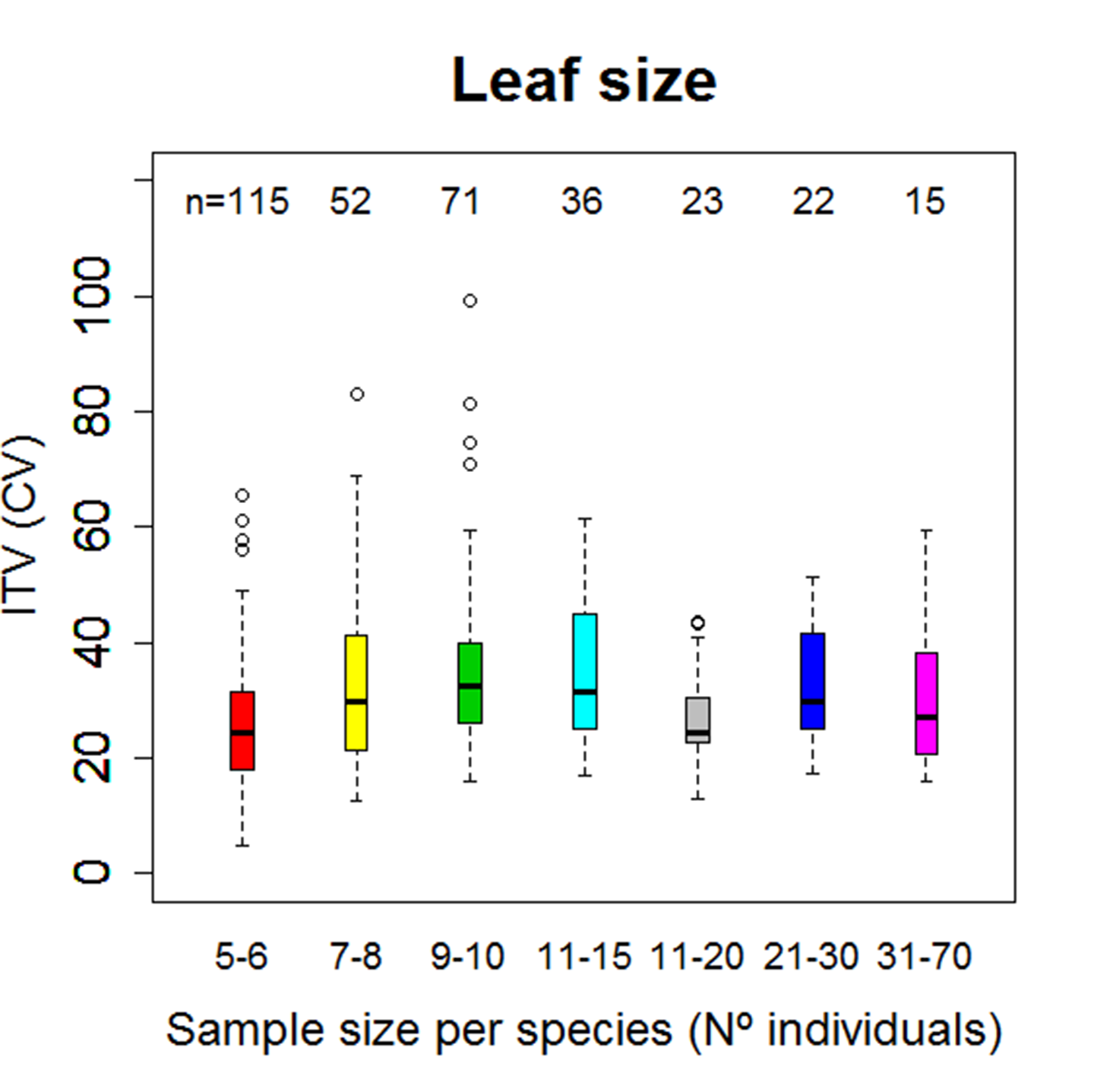

Supplement: S4 Fig — n: number of observations in each category. (TIF) [file pone.0172495.s005.tif]

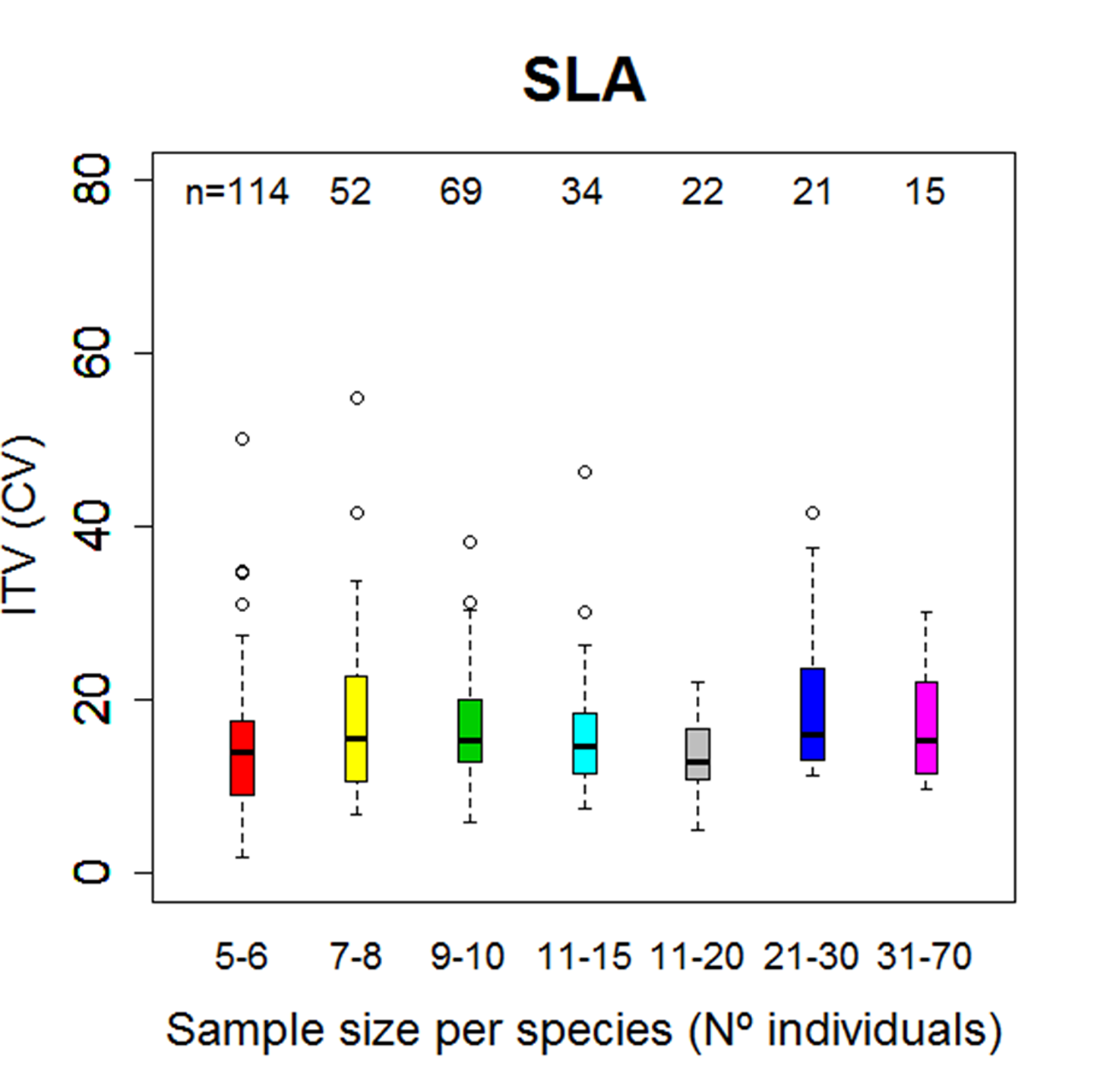

Supplement: S5 Fig — n: number of observations in each category. (TIF) [file pone.0172495.s006.tif]

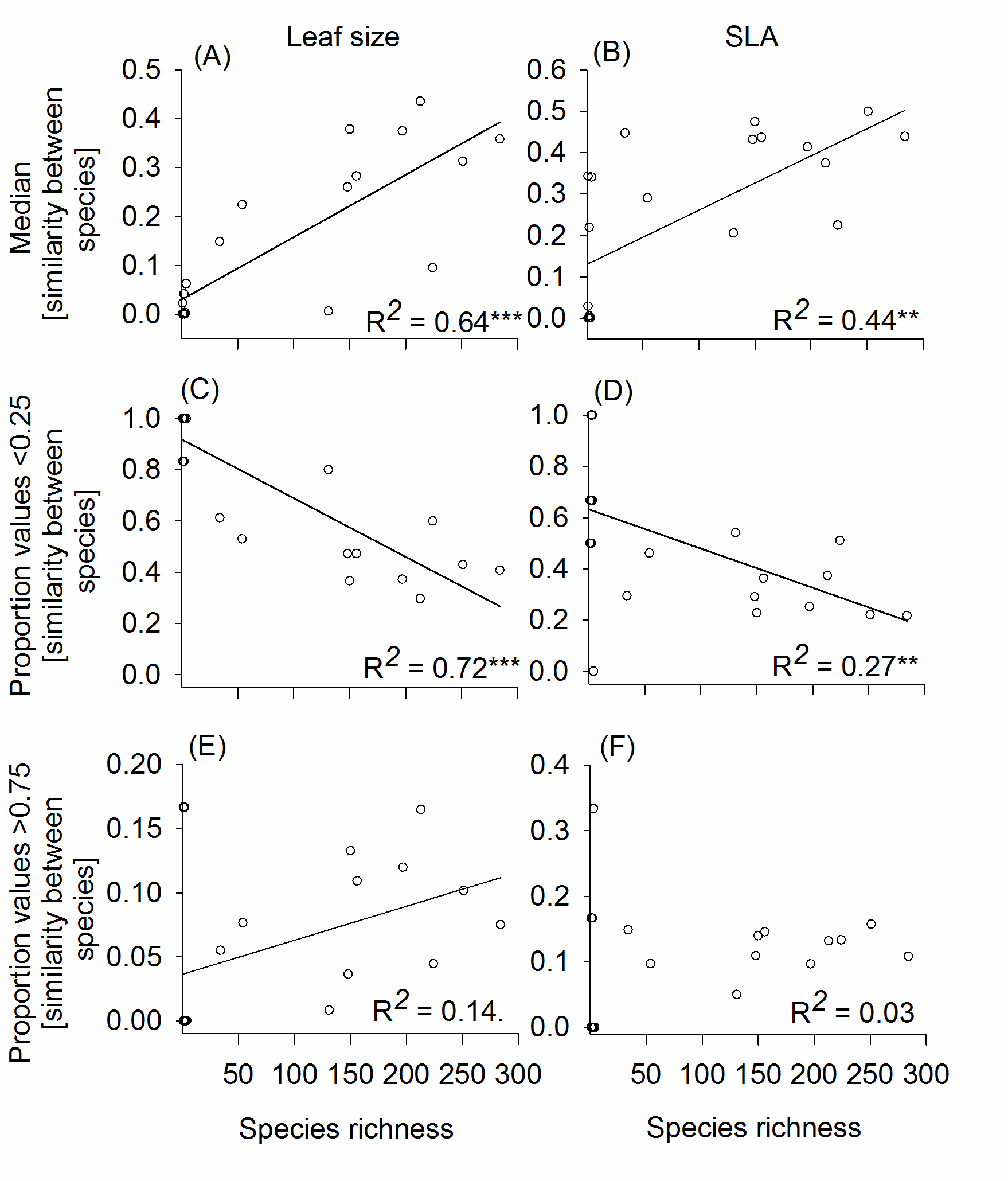

Supplement: S6 Fig — Linear regression models of the median values of trait overlap (panel A, B) and the proportion of low (less than 0.25; panel C, D) and high (> 0.75; panel E, F) values of degree of trait overlap between species for each forest community against species richness for both leaf size (left) and SLA (right). Trait similarity was calculated by kernel density approach using species with ≥ 5 individuals measured. (***): p-value <0.001; (**): p-value <0.01; (*): p-value < 0.05; (.): p-value <0.1. (TIF) [file pone.0172495.s007.tif]
